# Breast carcinomas with low amplified/equivocal HER2 by Ish: potential supporting role of multiplex ligation-dependent probe amplification

**DOI:** 10.1186/s13046-017-0613-2

**Published:** 2017-10-13

**Authors:** Cristiana Ercolani, Caterina Marchiò, Anna Di Benedetto, Alessandra Fabi, Letizia Perracchio, Patrizia Vici, Francesca Sperati, Simonetta Buglioni, Vincenzo Arena, Edoardo Pescarmona, Anna Sapino, Irene Terrenato, Marcella Mottolese

**Affiliations:** 1 Department of Pathology, Regina Elena National Cancer Institute, Via Elio Chianesi 53, 00144 Rome, Italy; 2 Department of Medical Sciences, University of Turin, Pathology Unit, Via Santena 7, 10126 Turin, Italy; 3 Medical Oncology 1, Regina Elena National Cancer Institute, Via Elio Chianesi 53, 00144 Rome, Italy; 4 Medical Oncology 2, Regina Elena National Cancer Institute, Via Elio Chianesi 53, 00144 Rome, Italy; 5 Biostatistic Unit, Regina Elena National Cancer Institute, Via Elio Chianesi 53, 00144 Rome, Italy; 6 Department of Pathology, Catholic University of Sacred Heart, Foundation Policlinico A. Gemelli, Rome, Italy; 7 Candiolo Cancer Institute - Fondazione del Piemonte per l’Oncologia (FPO), IRCCS, Str. Prov. 142, km 3.95, Candiolo, 10060 To, Italy

**Keywords:** Breast Cancer, HER2, Immunohistochemistry, *In Situ* Hybridization, MLPA

## Abstract

**Background:**

This is a retrospective cross sectional study aimed to verify whether Multiplex Ligation-dependent Probe Amplification (MLPA), a quantitative molecular assay, may represent a valuable reflex test in breast cancer with equivocal HER2 expression by immunohistochemistry and HER2 gene signals/nucleus (s/n) ranging between 4.0 and 5.9 by in situ hybridization.

**Methods:**

A series of 170 breast carcinomas scored as 2+ for HER2 expression by immunohistochemistry, were selected from our files and analyzed in parallel by silver in situ hybridization and by MLPA. According to ASCO-CAP 2013 guidelines, 54/170 tumors, displaying 4.0–5.9 HER2 gene s/n, were defined as low amplified (ratio ≥ 2) or equivocal (ratio < 2) on the basis of centromere enumeration probe 17 (CEP17) status. An independent set of 108 score 2+ breast cancers represented the external validation set. Concordance between the two techniques was assessed through the use of Cohen’s K statistic.

**Results:**

A concordance rate of 78.2% (Cohen’s K statistic: 0,548 95% CI:[0,419–0,677]) between in situ hybridization and MLPA was found in the whole series of 170 cases and of 55.5% (Cohen’s K statistic: −0,043 95% CI:[−0,271–0,184]) in the 54 tumors presenting 4.0–5.9 HER2 gene s/n. By MLPA, we found HER2 amplification or gain in 14% of the 21 BC presenting a disomic status and in 18% of the 33 BC presenting a CEP17 > 2.0. These data were further confirmed in the external validation set. Interestingly, the 54 low amplified/equivocal breast carcinomas presented a frequency of hormonal receptor positivity significantly higher than that observed in the amplified tumors and similar to the non-amplified one (*p* = 0.016 for estrogen receptor and *p* = 0.001 for progesterone receptor).

**Conclusions:**

To avoid to offer patients an ineffective therapy, HER2 status should be studied more thoroughly in low amplified and equivocal cases which can have lower response rates and shorter time to progression to trastuzumab. In this context, our data indicate that MLPA may be a reliable, objective supporting test in selecting HER2 positive breast cancer patients.

## Background

The introduction of trastuzumab and more recently of other anti HER2 treatments such as pertuzumab, and trastuzumab-emtansine (T-DM1) in early-stage and advanced HER2 positive breast cancer (BC) patients, has completely changed the natural history of the tumor both in terms of time to recurrence and survival [1–6]. Nowadays, to correctly identify HER2 positive BC patients who may benefit from these targeted therapies, is still a crucial clinical demand. In fact, a false-positive test could lead to an expensive ineffective treatment associated with potential side effects. Conversely, a false-negative test will deprive the patient of an important therapeutic option [7–9].

Immunohistochemistry (IHC) is the most common screening test used to detect HER2 protein expression in BC and may easily identify negative (score 0/1+) and positive (score 3+) cases whereas IHC 2+ equivocal results, occurring in up to a quarter of BC, need a reflex in situ hybridization (ISH) test to identify HER2 positive cases [10]. Moreover, the last ASCO-CAP guidelines [11], including incomplete membrane IHC positivity in the definition of score 2+, yielded an increase in the number of tumors, which need a reflex ISH test to confirm HER2 status [12, 13]. It has been widely reported that the majority of ISH tests, performed in IHC equivocal cases, may provide unambiguous positive (ratio > 2.0, average HER2 copy number ≥ 6.0) or negative (ratio < 2.0, average HER2 copy number < 4.0) results. However, it has been recently described [14] that approximately 5% of 2+ BC score present “non classical” HER2 amplification due to a ratio or a gene copy number close to the threshold. According to Ballard [14], the “non classical” amplified cases include (1) low amplified BC with a ratio ≥ 2.0 and an average HER2 copy number between 4.0 and 5.9, (2) co-amplified BC with a ratio < 2.0, but an average HER2 copy number ≥ 6.0, (3) monosomy-like BC with a ratio ≥ 2.0, but an average HER2 copy number < 4.0, (4) heterogeneous BC presenting clusters of amplified tumor cells (>10%) associated with clusters of non amplified tumor cells [11, 14]. This complex scenario is further complicated by ISH equivocal BC making up approximately 5% of cases which have an average HER2 copy number between 4.0 and 5.9, but a ratio < 2.0. In the latter case the ASCO-CAP guidelines 2013 recommend further HER2 testing on the same or other tumor samples. Nevertheless, the optimum HER2/CEP17 ratio cut-off for tumor response in BC patients treated with trastuzumab both in the (neo)-adjuvant and metastatic setting is still widely debated [15–19]. In view of this, novel emerging techniques are described and aimed to more accurately and more objectively estimate the HER2 status [20–26]. Although the majority of these novel techniques demonstrate a good overall correlation with each other in comparative studies, each assay has its own advantages and disadvantages and, up to now, there is neither real gold standard nor validated test to better define the equivocal subset. In this context, Multiplex Ligation-dependent Probe Amplification (MLPA), a high-throughput PCR-based technique, seems to provide an alternative valuable and cheap tool to more objectively define HER2 status. Aimed to validate its use in clinical practice, several authors [26–29] compared MLPA with conventional techniques [30, 31], obtaining optimal results mainly in clearly amplified and non amplified breast cancer. Conversely, limited MLPA data are available in the grey zone represented by BC with 4.0–5.9 HER2 gene s/n. For the purpose of our study, we evaluated 170 IHC HER2 2+ BC focusing on those cases showing 4.0–5.9 gene s/n to verify whether MLPA might be a useful reflex test in low amplified (ratio ≥ 2) and equivocal (ratio < 2) tumors by ISH. An independent series of 108 IHC HER2 2+ BC represented the external validation set.

## Methods

### Patients

The Pathology Department of the Regina Elena National Cancer Institute in-house analyzes all the BC with equivocal IHC results (score 2+) by ISH. Data from our internal dataset showed that in the last 8 years we found 3066 BC patients presenting a tumor with score 2+ by IHC. About 11% of the latter series presented 4.0–5.9 HER2 s/n. For the purposes of this retrospective cross sectional study, 170 BC scoring HER2 2+ by IHC were selected from our files. Our study comprised also an independent external set of 108 HER2 2+ BC patients provided by the Pathology Unit of the University of Turin (Italy) where all the analyses (IHC, FISH and MLPA) were performed. The two centers revaluated slides in order to confirm tumor histologic type, grading, percentage of tumor cells and HER2 equivocal expression based on ASCO-CAP guidelines 2013 in their own cases. Both centers successfully participated in regional quality control programs for the IHC assessment of HER2 [32, 33]. The study was reviewed and approved by the Local Ethic Committee at the Regina Elena National Cancer Institute and a written informed consent was obtained from all patients (del. n.180/2014).

### Immunohistochemistry

Three-micrometer sections of formalin-fixed paraffin-embedded breast cancers, were cut on SuperFrost Plus slides (Menzel-Gläser, Braunschweig, Germany). Estrogen (ER) and Progesterone (PgR) receptors were analyzed by using the monoclonal antibodies (MoAbs) 6F11 and 1A6 (Leica Biosystems, Italy) respectively. HER2 and Ki-67 were assessed by using the polyclonal antibody A0485 (PoAb, Dako, Milan, Italy) and the MoAb MIB-1 (Dako), respectively. Immunoreactions were revealed by a streptavidin-biotin enhanced immunoperoxidase technique (Super SensitiveMultiLink) in an automated autostainer (Bond III, Leica Biosystems). Diaminobenzidine was used as chromogenic substrate. The external center used the HercepTest preparation kit (Dako, Glostrup, Denmark, http://www.dako.com) and scored the results as previously described [27].

### IHC scoring criteria

HER2, hormonal receptor status and proliferation index (Ki-67) were obtained from the original pathology reports. In details, HER2 was scored according to the current guidelines [11]. Thresholds for estrogen receptor (ER), progesterone receptor (PgR) and Ki-67 proliferation index were ≥1%, >20% and ≥20, respectively [34].

### Dual in situ hybridization assay

To assess HER2 gene and chromosome 17 (CEP17) status we used a fully automated dual color in situ hybridization assay based on the use of an automated silver deposition technology (DDISH, Roche Diagnostic, Monza, Italy). All reagents included dinitrophenyl (DNP) HER2 and CEP17 probe cocktails. The ultraView SISH and ultraView Alkaline Phosphatase Red ISH detection kits were used. Slides were counterstained with hematoxylin and post-counterstained with a bluing reagent. After removal from the instrument, slides were rinsed with mild soap tap water, then rinsed in distilled water, dried at room temperature, and cover-slipped. The black HER2 SISH signals and the CEP17 red signals were enumerated in the tumor nuclei using a bright-field microscopy under a 100X oil immersion objective. After scanning the entire section, a total of 120 cells was analyzed in at least three selected fields.

### FISH assay

FISH was performed according to the manufacturer’s instructions with probes for HER2 and CEP17 (Abbott Molecular Diagnostics, Abbott Park, IL, https://www.abbottmolecular.com), as previously described [27, 35].

### ISH scoring criteria

According to the current ASCO-CAP guidelines, a BC displaying a ratio between HER2 gene and CEP17 ≥ 2.0 or with an average of more than or equal to six gene s/n is considered as amplified. The equivocal and the low amplified range is defined as HER2/CEP17 ratio < 2.0 and ≥2.0, respectively with an average gene copy number between 4.0 and 5.9 in both cases. All BC with a ratio < 2.0 and with an average < 4.0 gene s/n are designated as negative. An increased CEP17 copy number is considered to be present when a mean number of >2.0 s/n is observed in at least 60 tumor cell nuclei.

### Multiplex ligation-dependent probe amplification

Multiplex Ligation-dependent Probe Amplification (MLPA) (MRC-Holland, Amsterdam, The Netherlands) is a semi quantitative technique which needs only minute quantities of DNA isolated from paraffin-embedded tissues. Both participating centers performed MLPA as follows: invasive tumor areas as identified on serial haematoxylin/eosin (H&E) sections by a pathologist were harvested from 3 or 4 whole 5 μm thick paraffin sections by macrodissection. DNA was isolated using the QIAamp DNA kit (QIAGEN, Milan, Italy) according to the manufacturers’ instructions. This DNA solution, after centrifugation and quantification with NanoDrop TM 1000 spectrophotometer (ThermoScientific Fisher, Wilmington, DE, USA), was used in the MLPA. The P004-C1 ERBB2 kit (MRCHolland, Amsterdam, The Netherlands) contains 4 probes recognizing different sequences of the HER2 gene, and 27 probes for other genes on Chr17. In addition, 6 probes targeting genes on other chromosomes are included. Also the kit contains twelve reference probes specific for chromosomal regions, which have been found to be silent regions in CGH experiments. Briefly, 150–200 ng of target DNA per 5 μl of 10 mM pH 8.3 Tris-HCl 0.1 mM EDTA was denatured for 5 min at 98 °C after which 3 μl of the hemiprobe mix was added. The mixture was heated at 95 °C for 1 min and incubated at 60 °C overnight (16–20 h). After hybridization to the target sequences, the hemiprobes were ligated and then amplified. Ligation was performed with the temperature stable Ligase-65 enzyme (MRC-Holland) for 15 min at 54 °C. Next, the ligase was inactivated by heat in the thermocycler for 5 min at 98 °C and then paused at 20 °C. PCR was carried out for 35 cycles (30 s at 95 °C, 30 s at 60 °C, and 1 min at 72 °C). All tests were performed in duplicate on an Applied Biosystems® Veriti®Thermal Cycler. A variable stuffer sequence on one of the hemiprobes determined the length of the PCR product of each gene. The fragments obtained were analyzed with an ABI model 3130 capillary sequencer (Life technologies, Monza, Italy) using GeneScan™ 500 LIZ™ dye Size Standard (Life technologies, Warrington, UK).

Final results were calculated using dedicated software (Coffalyser.NET). The comparison with reference DNA samples derived from healthy individuals is essential in obtaining an accurate final result. To this end three negative reference samples (normal breast) were taken along in each MLPA run to normalize MLPA ratios. For genes with more than one probe present in the kit, the mean of all the probe peaks of this gene in duplicate was calculated. Mean values <0.7 refer to gene deletion, between 0.7–1.3 to normal gene status and values between 1.3–1.5 and >1.5 to gains and gene amplification respectively. These cut-off were validated in breast cancer cell lines showing HER2 amplification (BT474: 6 HER2 copy number by FISH, MLPA ratio of 5), HER2 gain (T47D: 4 HER2 copy number by FISH, MLPA ratio of 1.32) and lacking HER2 gain or amplification (MCF7: 2 HER2 copy number by FISH, MLPA ratio of 0.61).

### Statistical analysis

All variables of interest were summarized through frequencies and percentage values. Concordance between the two techniques was assessed through the use of Cohen’s K statistic (K) [36]. Each K value was reported with its relative 95% Confidence interval (95% CI) and interpreted in a qualitative manner based on the Landis and Koch classification criteria [37].

## Results

### Patient characteristics

According to the World Health Organization classification [38] out of the 170 BC with HER2 score 2+ by IHC included in the study, 161 were infiltrating carcinomas of no special type, 4 invasive lobular carcinomas and 5 special histologic types. As summarized in Table 1, 12 (7%) tumors were graded, using the Elston and Ellis scoring system [39], as well differentiated (G1), 76 (45%) and 82 (48%) as moderately (G2) and poorly differentiated (G3) carcinomas, respectively. ER and/or PgR were positive in 132 (78%) BC and Ki67 was high in 97 (57%) cases. Out of the 170 BC analyzed by DDISH, 61 (36%) presented a HER2 s/*n* < 4.0, 54 (32%) between 4.0–5.9 s/n and 55 (32%) ≥6.0 s/n. Of the 108 score 2+ BC, belonging to the external validation set, 92 were infiltrating carcinomas of no special types, 8 invasive lobular carcinomas and 8 were special histologic types. Seventeen (16%) tumors were graded as well differentiated (G1), 55 (51%) and 26 (24%) as moderately (G2) and poorly differentiated (G3) carcinomas, respectively. ER and/or PgR were positive in 99 (92%) BC and Ki67 was high in 52 (48%) cases. FISH evidenced 80 (74%) BC with <4.0 HER2 s/n, 10 (9%) with 4.0–5.9 HER2 s/n and 18 (17%) with HER2 ≥ 6.0 s/n.

**Table 1:** Biopathological characteristics of the two breast cancer sets

	Internal set	External set
Total number of patients	170	108
Grading		
1	12 (7%)	17 (16%)
2	76 (45%)	55 (51%)
3	82 (48%)	26 (24%)
unknown	0 (0%)	10 (9%)
Node		
Negative	67 (39%)	55 (51%)
Positive	77 (46%)	38 (35%)
unknown	26 (15%)	15 (14%)
HR Status		
ER and/or PgR positive	132 (78%)	99 (92%)
ER /PgR negative	38 (22%)	9 (8%)
Ki67		
High ≥20%	97 (57%)	52 (48%)
Low < 20%	73 (43%)	56 (52%)
HER2 gene s/n		
<4.0	61^a^ (36%)	80^b^(74%)
4.0–5.9	54^a^(32%)	10^b^ (9%)
≥6.0	55^a^ (32%)	18^b^(17%)

*HR* Hormonal Receptor, *ER* Estrogen Receptor,*PgR* Progesterone Receptor, *s/n* signals/nucleus

^a^Silver In Situ Hybridization; ^b^Fluorescence In Situ Hybridization

### Concordance between ISH and MLPA results in the internal and in the validation set

A concordance rate of 78.2% (Table 2, Cohen’s K statistic: 0.548 95% CI [0.419–0.677]) between ISH and MLPA was found in the whole series of 170 cases. Overlapping results were found in the external set of 108 BC where the concordance rate was 76.9% (Cohen’s K statistic: 0.464 95% CI [0.280–0.648]). In each series, the concordance value between the two techniques, showed a moderate agreement (0.41 < k < 0.60) following the Landis and Koch criteria. To understand which variables may affect this agreement, we subdivided our series and the external validation series into 2 separate groups as follow: (1) 61 and 79 clearly non amplified BC (<4.0 HER2 s/n) as well as 55 and 19 highly amplified BC (≥6.0 HER2 s/n). (2) 54 and 10 BC presenting an equivocal number of gene s/n (4.0–5.9 HER2 s/n).

**Table 2:** Concordance between ISH and MLPA in the internal and external validation set

	DDISH (*N* = 170)*		Cohen’s K Statistic [95% CI]
	NA	A	
	94	76	
MLPA			0.548 [0.419–0.677]
NA	86	29	
A	8	47	
	FISH (*N* = 108)**		
	NA	A	
	87	21	
MLPA			0.464 [0.280–0.648]
NA	64	2	
A	23	19	

*NA* Non Amplified, *A* Amplified, *MLPA* A = Amplified + Gain

* Internal set ** External set

### First group analyses

**Fig. 1:**
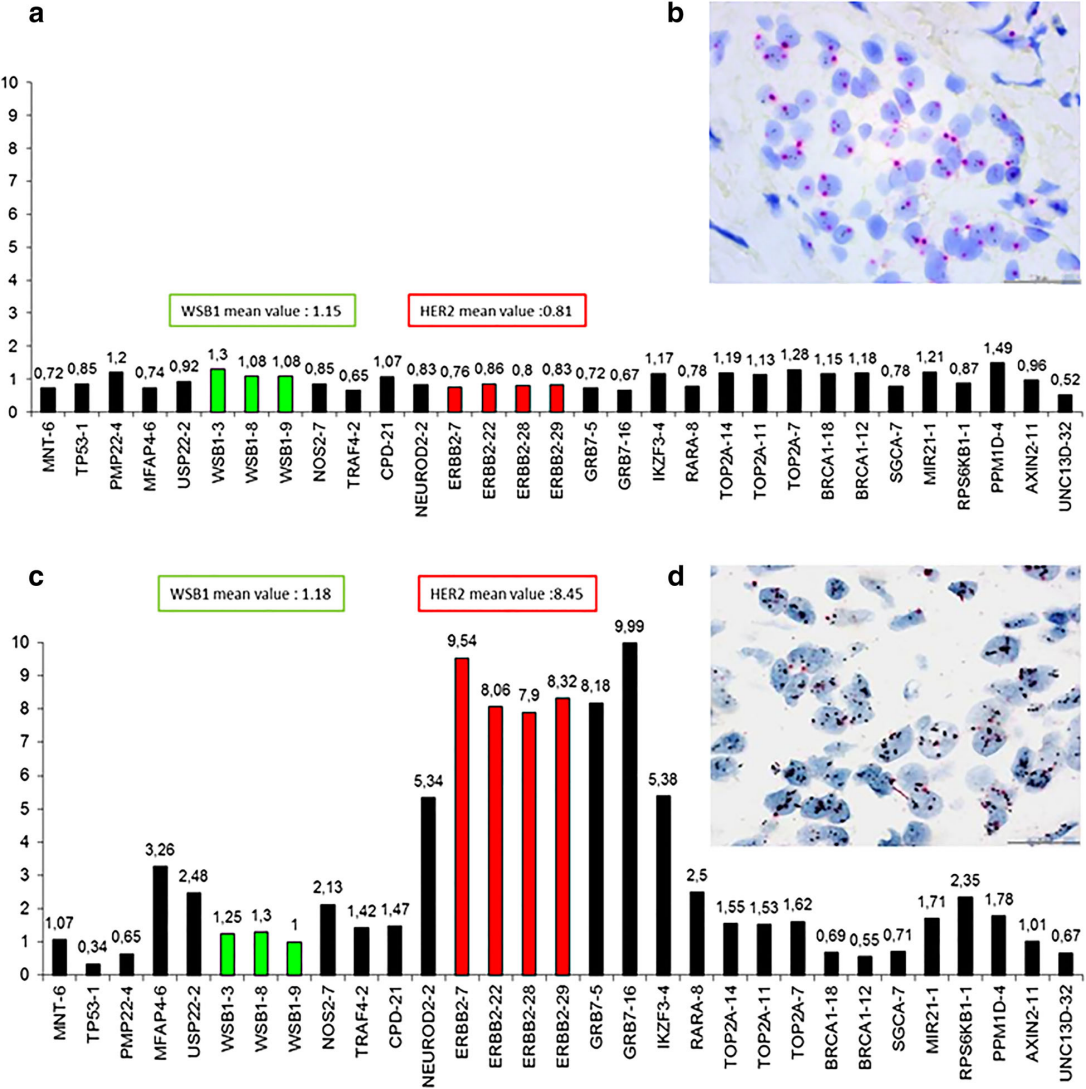
MLPA and DDISH in HER2 non amplified and amplified breast carcinomas. Two exemplificative BC **a**-**d** showing respectively a HER2 normal gene status **a** by MLPA (mean value 0.82) and **b** by DDISH (ratio < 2.0) and HER2 gene amplification **c** by MLPA (mean value 8.45) and **d** by DDISH (>6.0 s/n, ratio ≥ 2.0). Scale bar = 30 μm

Table 3 summarizes the concordance between ISH and MLPA both in the non amplified (NA) and amplified (A) cases in our series and in the external validation set. In our series, we found that of the 61 HER2 NA cases by DDISH, 59 (97%) were also non amplified by MLPA (Fig. 1a, b). Among the 55 HER2 amplified BC by DDISH, MLPA confirmed HER2 gene amplification in 41 cases (75%) (Fig. 1c, d). In contrast, 11 tumors (20%) had a normal HER2 gene status and 3 (5%) presented a HER2 gene gain. A concordance rate between DDISH and MLPA of 88,8% (Cohen’s K statistic: 0,773 95% CI:[0,594–0,953]) was found. Following Landis and Koch criteria, this value showed a substantial agreement (0.61 < k < 0.80) between the two techniques. Concerning the external validation set, among the 79 NA cases, MLPA confirmed the lack of amplification in 62 tumors (79%). In the remaining 17 BC, 7 (9%) presented a HER2 gain and 10 (12%) were amplified. Among the 19 HER2 amplified BC by FISH, MLPA confirmed HER2 gene amplification in 15 tumors (79%), 2 (10.5%) had a normal HER2 gene status and 2 (10.5%) presented a gain. The concordance rate between FISH and MLPA was of 80.6%, Cohen’s K statistic: 0.523 95% CI:[0,339–0,706]. Following the Landis and Koch criteria, this value shows a moderate agreement (0.41 < k < 0.60) between the two techniques.

**Table 3:** ISH and MLPA results in High Amplified/Non Amplified breast cancers in the internal and external validation set

	DDISH (*N* = 116)^a^		FISH (*N* = 98)^b^	
	gene s/n		gene s/n	
	<4.0	≥6.0	<4.0	≥6.0
	61	55	79	19
MLPA				
NA	59 (97%)	11 (20%)	62 (79%)	2 (10.5%)
GAIN	0 (0%)	3 (5%)	7 (9%)	2 (10.5%)
A	2 (3%)	41 (75%)	10 (12%)	15 (79%)

*NA* Non Amplified, *A* Amplified, *NA* < 1.3 mean value, *GAIN* ≥ 1.3 < 1.5 mean value, *A* ≥ 1.5 mean value

^a^Internal set ^b^External set

### Second group analyses

**Fig. 2:**
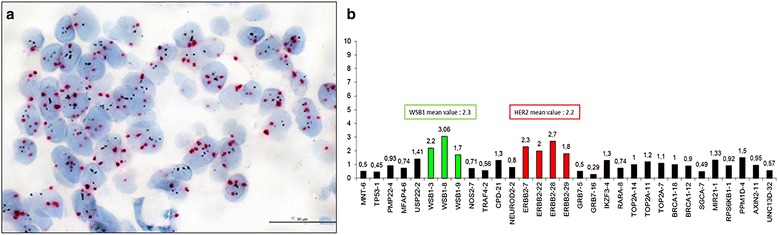
An equivocal HER2 breast cancer by DDISH and MLPA. An exemplificative case of breast cancer HER2 equivocal **a** by DDISH (gene s/n of 5.2 and CEP17 > 2.0) and **b** amplified by MLPA (mean value of 2.2). The tumor presents a co-amplification of the CEP17 gene WSB1 (mean value 2.3). Scale bar = 30 μm

As summarized in Table 4, we focused on the 54 BC with 4.0–5.9 HER2 s/n belonging to our series and we found that 21 (39%) cases presented a normal CEP17 status and 33 (61%) had an increased CEP17 s/n by DDISH. According to the 2013 ASCO-CAP, the 21 disomic BC could be considered amplified (ratio ≥ 2.0), whereas the 33 BC presenting an altered CEP17 status (>2.0 s/n) were equivocal and need a reflex test to definitely establish HER2 status. MLPA is a method capable of highlighting, beyond HER2, also alterations of CEP17 genes including WSB1 and NOS2A loci which are closed to the centromeric region. Out of the 21 BC presenting 2 CEP17 s/n, 1 (5%) was HER2 amplified (mean value ≥1.5) by MLPA, 2 (9%) showed a gain (mean value ≥1.3 but <1.5), the other 18 resulted HER2 non amplified (mean value <1.3). Among the 33 equivocal cases due to an altered CEP17 status by DDISH, MLPA found 4 (12%) HER2 amplified BC (mean value ≥1.5, Fig. 2), 2 (6%) with a gain (mean value ≥1.3, but <1.5) and the remaining 27 non amplified (mean value <1.3) In the latter cases, we found a 55,5% concordance rate between DDISH and MLPA (Cohen’s K statistic: -0.043, 95%CI:[−0,271–0,184]). Therefore, no agreement was observed.

**Table 4:** ISH and MLPA results in low amplified and equivocal breast cancers in the internal and external set

	DDISH (*N* = 54)^a^		FISH (*N* = 10)^b^	
	4.0–5.9/=2	4.0–5.9/>2	4.0–5.9/=2	4.0–5.9/>2
	21	33	2	8
MLPA				
NA	18 (86%)	27 (82%)	0 (0%)	2 (25%)
GAIN	2 (9%)	2 (6%)	1 (50%)	2 (25%)
A	1 (5%)	4 (12%)	1 (50%)	4 (50%)

*NA* Non Amplified, *A* Amplified, *NA* < 1.3 mean value, GAIN ≥1.3 < 1.5 mean value, A ≥ 1.5 mean value

^a^Internal set: gene /CEP17 s/n ^b^External set: gene /CEP17 s/n

In the external validation series, out of the 10 BC with 4.0–5.9 HER2 s/n by FISH, 2 presented a disomic pattern of which, through MLPA, 1 (50%) was amplified (mean value ≥1.5) and 1 (50%) showed a gain (mean value ≥1.3 but <1.5). Among the 8 cases presenting an altered CEP17 status (>2.0), MLPA found 4 BC (50%) HER2 amplified (mean value ≥1.5), 2 (25%) with a gain (mean value ≥1.3 but <1.5) and the other 2 (25%) non amplified (mean value <1.3). The concordance rate between FISH and MLPA was 40% (Cohen’s K statistic: 0,118 95%CI:[−0,174–0,409]). Following the Landis and Koch criteria, this value shows only a slight agreement (0.01 < k < 0.20) between the two techniques. We found amplification or gain of WSB1 and/or NOS2A in 16/33 cases (48.5%) of our series (data not shown) and in 5/8 (62.5%) cases of the external validation series.

### Association between HER2 gene copy number and immunophenotypical factors in the 54 low amplified and equivocal breast cancers

**Fig. 3:**
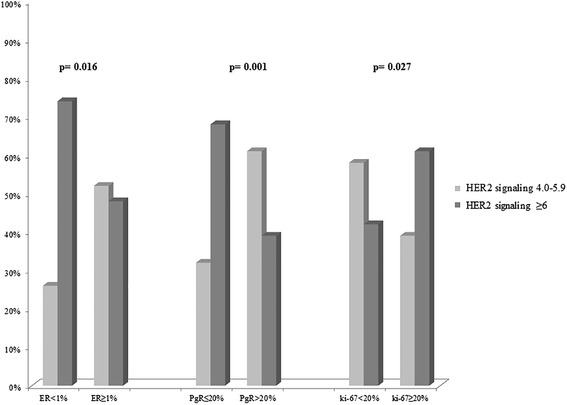
Association between HER2 s/n and immunophenotypical factors. The 64 breast carcinomas presenting 4.0–5.9 HER2 s/n included in our study have a significantly higher percentage of estrogen (p = 0.016) and/or progesterone (*p* = 0.001) positivity, and a lower percentage of Ki67 proliferation index (*p* = 0.027) than highly amplified tumors (≥6.0 HER2 s/n)

We explored the potential association between the HER2 gene copy number (<4.0, between 4.0–5.9, ≥6.0) and immunophenotypical factors ER, PgR and Ki67 in the entire cohort of 278 BC. Figure 3 shows that BC presenting a low amplification rate (4.0–5.9 HER2 s/n, CEP17 = 2.0) have a significantly higher percentage of ER (*p* = 0.016) and/or PgR positivity (*p* = 0.001) and a lower percentage of Ki67 proliferation index (*p* = 0.027) than highly amplified tumors (>6.0 HER2 s/n). In particular, BC with 4.0–5.9 HER2 gene s/n show an immunophenotypical profile more similar to the HER2 non amplified BC than amplified BC (*p* = 0.260, *p* = 0.257, *p* = 0.947) (data not shown).

## Discussion

Our Pathology Department analyzes HER2 status in more than 1000 BC per year. In particular, between 2009 and 2016, 3066 consecutive cases showed IHC HER2 equivocal status (score 2+) and underwent ISH analysis to definitely identify HER2 positive BC. Even though in the majority of cases, ISH testing is able to accurately discriminate amplified from non-amplified tumors, 11% of our BC routine series presented a mean of HER2 gene s/n ranging between 4.0 and 5.9. According to the 2013 ASCO-CAP guidelines [11], 8% were amplified due to a HER2/CEP17 ratio ≥ 2.0 and 3% were equivocal since CEP17 signals produced a ratio < 2.0. The two categories, low amplified and equivocal, as highlighted in our routine series, are in line with those reported by Ballard et al. [14] in a recent and elegant study concerning the non classical HER2 FISH results. It is of paramount clinical importance to carefully define the HER2 status in these uncommon subsets of BC in which the mean CEP17 copy number drives in the final classification based on the ISH algorithm proposed by the guidelines. To this end, we evaluated whether MLPA, a quantitative molecular method [26, 28, 29, 40] may be a helpful reflex test to define the HER2 status in 54 BC presenting a non classical HER2 ISH results (4.0–5.9 HER2 gene s/n). One hundred and sixteen BC including 55 HER2 clearly amplified (>6.0 gene s/n) and 61 clearly non amplified (less than 4.0 gene s/n, ratio < 2.0) BC represented our control series. Moreover, an independent set of 108 IHC 2+ BC, of which 10 had a non classical HER2 gene pattern, represented our external validation set. Several studies already demonstrated that MLPA is an easy method to perform and interpret HER2 status with a good correlation with IHC and ISH [28–31]. Nevertheless, to the best of our knowledge, this is the first study which investigated whether MLPA may provide useful diagnostic information in two independent BC series presenting a mean of 4.0–5.9 HER2 genes/n which can be often difficult to define as amplified or not. The concordance rate between DDISH and MLPA obtained in this series of 54 BC was 55.5%, a percentage significantly lower than that obtained in the 116 BC made up by clearly amplified and non amplified BC (concordance 88.8%). This observation further indicates the peculiar biological characteristics of these low amplified or equivocal cases. As already reported by Ballard [14], we observed, in fact, that tumors harboring 4.0–5.9 HER2 gene s/n had a frequency of ER positivity similar to the non amplified category. These data were confirmed in the external validation set supporting the recommendation to consider all the clinico-pathological features on a case-by-case basis when planning HER2-targeted therapies in these critical cases. Several recent studies which compared MLPA and ISH techniques, were broadly concordant with our results. The data reported so far have demonstrated that there are specific scenarios in which MLPA may be of particular value in HER2 testing when selecting patients to undergo anti HER2 treatment [26–28, 40]. In this context, BC patients displaying a mean of the HER2 gene s/n ranging between 4.0–5.9, including both low amplified and equivocal tumors, could benefit most from MLPA. We have previously reported a small cohort of HER2 equivocal BC where 75% presented a normal HER2 status whereas 25% showed HER2 gain [27]. Here we now report data on a series of 54 low amplified and equivocal cases where MLPA uncovers HER2 amplification in about 17% of cases and seems to be an optimal reflex test mainly in the group of 33 cases defined equivocal by DDISH. The external validation series had a strikingly similar frequency. Whether patients with HER2 equivocal tumors should receive targeted therapy remains a challenging question for oncologists, particularly as reporting guidelines evolve. The 2007 ASCO-CAP guidelines [41] did not recommend anti HER2 treatment for patients with an equivocal test. On the other hand, the 2013 ASCO/CAP guidelines [11] opened the possibility to oncologists to consider HER2-targeted therapy also for patients with equivocal HER2 test results, even after reflex testing with an alternative assay. Clearly, there is a need to further examine this critical subset of tumors, whose relative frequency has increased following the application of the 2013 ASCO-CAP guidelines [11]. A recent re-evaluation of HERA trial FISH results according to the 2013 ASCO-CAP guidelines on 6018 BC, showed an increase of equivocal cases from 0.7% to 1.9% [9]. In another recent study [42], the authors reviewed a consecutive series of 904 BC observing a switch from HER2 FISH negative in HER2 FISH equivocal in 7,3% of cases. The majority of these reclassified cases presented a gained CEP17. The lack of an overall agreement between ISH and MLPA assays, mainly in tumors with 4.0–5.9 HER2 gene s/n, is due to both genetic heterogeneity and tumor cellularity of the sample [27]. In line with other authors [28], we observed that the best correlation between the two techniques is found when there is more than 30% of cancer cells in the test sample. Therefore, in order to eliminate any potential bias in our results due to low tumor cell percentage, the 170 BC included in the present study had to have at least 30% of tumor cells in the entire section. Furthermore, a tumor macrodissection was performed before MLPA testing. All cases belonging to the second group of 54 BC had the same range of mean HER2 gene s/n (4.0–5.9), but varied in the mean CEP17 s/n. According to 2013 ASCO-CAP, all the disomic tumors were HER2 amplified by ISH whereas MLPA found HER2 amplification or gain in 14% of the latter cases. In the group of equivocal tumors MLPA may objectively identify HER2 amplification in 18% of BC. This data raises the question whether the lack of amplification by MLPA is due to a bias induced by the method itself or vice-versa MLPA was able to exactly identify only the fraction of the true amplified cases. Concerning the first issue, the lack of amplification by MLPA may be due to intermixed genetic heterogeneity, an important biological mechanism that correlates with a low level of amplification and equivocal HER2 status. Lee et al. [43] analyzed 443 HER2 positive BC and found HER2 regional and genetic heterogeneity in 6.2% and 6.8%, respectively. These authors demonstrated that both types of heterogeneity were significantly associated with low levels of HER2 gene amplification. The objective results provided by MLPA in identifying the HER2 status may be of particular clinical importance. Recent studies indicated, in fact, that patients with overall low-level or equivocal HER2 amplification had significantly lower response rate to neo-adjuvant trastuzumab [44] and shorter time to progression and overall survival in metastatic BC treated with trastuzumab based chemotherapy than did those with high-level amplification [18, 19]. Qian et al. [45] compared the HER2 FISH status based on the 2007 and 2013 ASCO/CAP guidelines in 1931 BC cases. The authors noted that the last guidelines, although improving the identification of amplified cases, provided an increase in the equivocal cases. Furthermore, the observation that FISH equivocal cases do not always reflect HER2 overexpression might suggest the growing need to carefully select patients who can benefit from HER2 targeted treatment. These concerns were widely debated during the 15th St Gallen International Breast Cancer Conference [46]. A randomised phase III trial, NSABP B47 is ongoing and will probably elucidate the best treatment approach for these patients.

## Conclusions

In this study we confirmed that MLPA is a valuable and reproducible method to identify HER2 status in clearly non amplified or high amplified breast carcinomas. Nevertheless, the benefit of HER2-targeted therapies for BC patients presenting an equivocal/low level HER2 status are currently unknown, as assessed by the panelists partecipating to the 15th St Gallen International Breast Cancer Conference. These observations strengthen the concept that HER2 status should be studied more thoroughly in low amplified and equivocal cases avoiding to offer patients an ineffective therapy. In this context, our data indicate that MLPA could be an alternative, objective supporting test in selecting HER2 positive breast cancer patients. Nevertheless, our data should be considered as preliminary results to be further validate on a larger and prospective number of cases.

## Abbreviations

BC: Breast Cancer
CEP17: Centromere Enumeration Probe 17
DDISH: Dual Silver *In Situ* Hybridization
ER: Estrogen Receptor
FISH: Fluorescence *In Situ* Hybridization
IHC: Immunohistochemistry
ISH: *In Situ* Hybridization
MLPA: Multiplex Ligation-dependent Probe Amplification
NA: Non Amplified
PgR: Progesterone Receptor
s/n: Signal/nucleus
